# Hydropathy Landscapes of Histone–DNA Interactions in Chromatin Building Blocks

**DOI:** 10.1021/acs.jpcb.6c00520

**Published:** 2026-03-27

**Authors:** Ratnakshi Mandal, Andre Christophe Noel, Anna M. Baur, Shikha Nangia

**Affiliations:** Department of Biomedical and Chemical Engineering, 2029 Syracuse University, Syracuse, New York 13244, United States

## Abstract

Histone–DNA interactions define the fundamental building blocks of chromatin, yet their physicochemical organization is most often interpreted through structural and electrostatic descriptors. Here, we apply the Protocol for Assigning a Residue’s Character on a Hydropathy (PARCH) scale to multiple aspects of the nucleosome that are critical for DNA packaging, providing a residue-resolved, environment-dependent measure of hydrophobic and hydrophilic character. Using PARCH, we show that nucleobase hydropathy remains largely invariant upon nucleosome formation, whereas the DNA backbone undergoes a pronounced quantitative redistribution, yielding a bimodal hydropathy profile that distinguishes histone-contacting from solvent-exposed regions and arises from periodic histone–DNA contacts and conserved arginine anchors. Extending this framework, we demonstrate that histone core hydropathy is strikingly conserved across species that higher-order assembly into dinucleosomes—key structural intermediates in chromatin fiber formation—selectively reduces DNA backbone hydrophilicity at buried internucleosomal interfaces without altering histone hydropathy, and that the nucleosomal acidic patch remains largely hydropathy-insensitive to DNA wrapping. Finally, we show that cytosine methylation selectively decreases the DNA backbone hydrophilicity, providing a quantitative physicochemical mechanism for enhanced nucleosome stability. Taken together, these results illustrate how small, localized hydropathy modulations at histone–DNA interfaces can have outsized impacts on DNA packaging and chromatin organization and position PARCH as a powerful quantitative framework for mapping physicochemical landscapes across chromatin building blocks and epigenetically regulated states.

## Graphical abstract

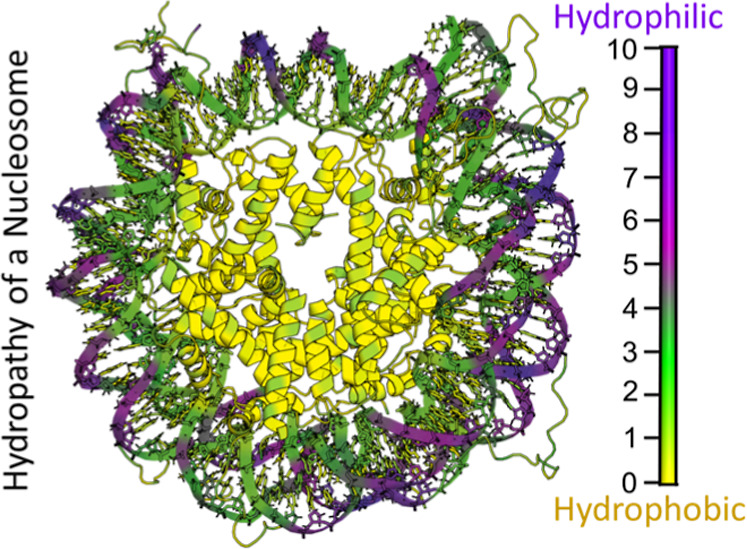

## Introduction

Chromatin organization arises from a complex interplay of molecular interactions that govern how DNA is packaged, stabilized, and dynamically accessed within the nucleus.^1–3^ While histone–DNA interactions have traditionally been interpreted through structural geometry and electrostatics,^4–6^ an essential physicochemical dimension, hydropathy, has received comparatively little attention. Hydropathy governs how biological macromolecules interact with water and ions, shaping local hydration, interfacial energetics, and molecular stability.^7,8^ In heterogeneous and multiscale systems such as chromatin, where DNA and proteins continuously transition between buried and solvent-exposed environments, hydropathy is expected to play a central role in determining how DNA packaging is achieved and regulated. However, a residue-resolved and environmentally dependent description of hydropathy in chromatin has been lacking.

The nucleosome, the fundamental building block of chromatin, provides an ideal framework for interrogating hydropathy at protein–DNA interfaces. In eukaryotes, approximately 147 base pairs of double-helical DNA wrap around a histone octamer composed of two copies each of H2A, H2B, H3, and H4.^4,9^ This histone core–DNA shell architecture enables meters of genomic DNA to be compacted into a micron-scale nucleus while preserving the dynamic accessibility required for transcription, replication, and repair.^10,11^ Achieving this balance requires precise modulation of DNA bending, hydration, and interfacial energetics, all of which are inherently linked to hydropathy.

High-resolution X-ray crystallography established the structural framework of the nucleosome and revealed the dominant role of electrostatics, with positively charged histone residues neutralizing the negatively charged DNA backbone and stabilizing sharp DNA curvature.^4–6^ Cryo-electron microscopy has since expanded this view by capturing nucleosomes in multiple conformational states and higher-order assemblies, including dinucleosomes and chromatin fibers.^12–14^ These studies demonstrate that chromatin is intrinsically dynamic, exhibiting nucleosome breathing, linker DNA flexibility, and structural heterogeneity that are essential for genome regulation. However, structural descriptions alone do not fully capture the physicochemical environment experienced by DNA and histones as they transition between buried and solvent-exposed states.

Comparative analyses across species further show that the nucleosome architecture is remarkably conserved from yeast to humans. Core histones share high sequence identity and nearly identical three-dimensional folds, reflecting strong evolutionary constraints on histone–DNA interactions.^6,10^ At the same time, subtle variations in residue composition, surface chemistry, histone variants, and post-translational modifications^15–17^ modulate chromatin stability and regulatory potential in species-specific ways. How these variations translate into altered local hydration and interfacial energetics at the histone–DNA interface remains poorly understood.

Beyond individual nucleosomes, higher-order chromatin organization depends critically on interactions between neighboring nucleosomes. Dinucleosomes, consisting of two nucleosomes connected by linker DNA, represent the simplest higher-order chromatin assembly and a key intermediate in chromatin fiber formation.^13,18^ Structural and biochemical studies show that dinucleosomes adopt multiple relative orientations stabilized by histone tail interactions, acidic patch,^19,20^ arginine contacts, and linker DNA geometry. These assemblies introduce spatial asymmetry, creating buried internucleosomal interfaces alongside solvent-exposed regions, suggesting that hydropathy redistribution may be a fundamental driver of chromatin compaction.^21,22^

Epigenetic modifications further modulate chromatin organization by altering the DNA physicochemical properties. Cytosine methylation at the 5-position of the pyrimidine ring introduces hydrophobic character into DNA and has been shown to increase nucleosome stability, reduce DNA breathing, and promote chromatin compaction without altering nucleosome core structure.^23–25^ These effects are often attributed to changes in DNA shape and electrostatics, yet their hydropathy consequences have not been quantitatively resolved.

Here, we address these gaps by mapping hydropathy landscapes of histone–DNA interactions across chromatin building blocks using the Protocol for Assigning a Residue’s Character on a Hydropathy scale, PARCH, which provides a unified, residue-resolved, and environment-sensitive measure of hydrophobic and hydrophilic character that captures nanoscale topographical effects rather than relying on fixed residue-based scales.^26–29^ This framework is uniquely suited to chromatin, where local environment, solvent exposure, and molecular context vary continuously.^28^ Figure 1 provides an integrated schematic overview of the distinct but interconnected physicochemical mechanisms through which hydropathy is modulated across nucleosomes and higher-order chromatin assemblies, framing the analyses presented in this work.

**Figure 1:**
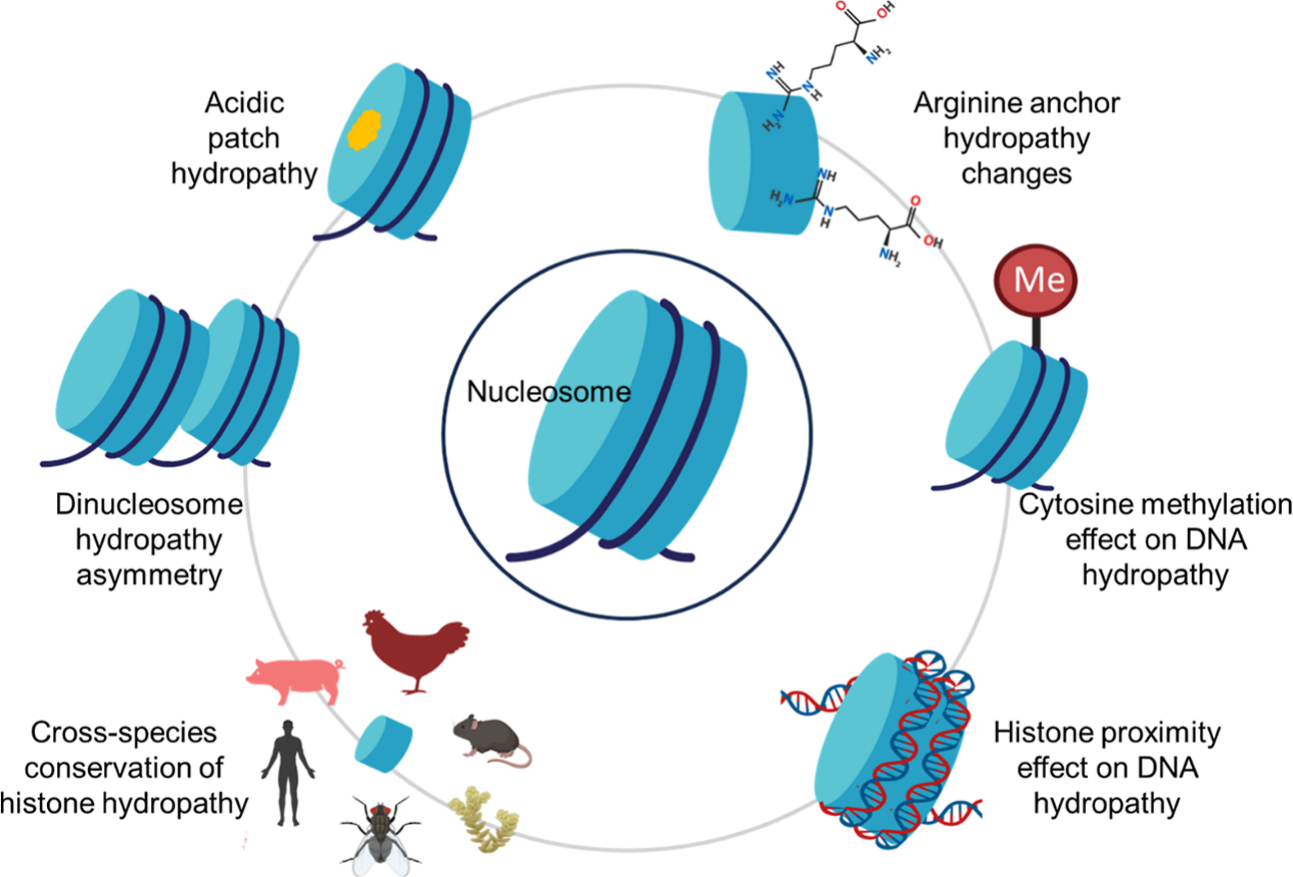
Integrated schematic of nucleosome hydropathy modulation. Six physicochemical mechanisms influencing DNA and histone hydropathy: (1) histone proximity alters DNA backbone hydration; (2) arginine anchors reduce local hydrophilicity via minor groove insertion; (3) acidic patch residues maintain stable hydropathy despite DNA wrapping; (4) dinucleosome formation induces asymmetric backbone hydration; (5) cytosine methylation decreases DNA backbone hydrophilicity; and (6) nucleosome–nucleosome interfaces selectively modulate DNA solvation. These effects collectively shape chromatin stability, accessibility, and regulatory potential.

Our results reveal that chromatin organization is accompanied by systematic and localized redistribution of hydropathy rather than global changes in structure or chemistry. By defining hydropathy landscapes across nucleosomes, dinucleosomes, species, and epigenetic states, this work establishes hydropathy as a fundamental physicochemical determinant of DNA packaging and provides a unifying framework for understanding how small, local interactions can produce large-scale effects on genome organization.

## Methods

### System Preparation

Our data set consists of 22 nucleosomes across multiple species and one dinucleosome. The nucleosome structures were obtained from the Protein Data Bank in legacy PDB format (Table S1). Each system was placed in a cubic simulation box and solvated with TIP3P^30^ water molecules, with the required number of counterions added to neutralize the system and NaCl included to achieve a salt concentration of 0.15 M. Energy minimization was performed using the steepest descent algorithm,^31^ followed by a two-step equilibration protocol. First, each system was equilibrated for 100 ns under isothermal–isochoric (NVT) conditions at 300 K using the velocity-rescale thermostat.^32^ This was followed by a second equilibration for 100 ns under isothermal–isobaric (NPT) conditions at 1 bar using the Berendsen barostat,^33^ with positional restraints of 1000 kJ mol^–1^ nm^–2^ applied to heavy atoms. Finally, a 100 ns production molecular dynamics simulation was carried out under the same NPT conditions using the Parrinello–Rahman barostat^34^ with all position restraints removed.

### PARCH Scale Simulations

To perform the PARCH scale calculations, the histone–DNA complexes were first fully relaxed without positional restraints to allow interfacial contacts and solvent organization to equilibrate. PARCH simulations were then performed for all systems. The details of the PARCH method have been described previously;^26,28^ a summary is provided here. Each system was annealed from 300 to 800 K at a heating rate of 0.1 K ps^–1^ over a total duration of 5 ns. During this annealing process, the nucleosome was position-restrained with a force constant of 10,000 kJ mol^–1^ nm^–2^ to prevent conformational changes. The parameters used for these calculations are provided in Table S2 in the Supporting Information.

After completion of the PARCH analysis, hydropathy values were written to the nucleosome PDB file as an additional column, with two values assigned for DNA—backbone (BB) and nucleobases (NB)—and one value assigned for protein residues. PyMOL^35^ was used to create illustrations. Jalview^36^ was used to align amino acid sequences and generate illustrations.

### Methylation

Site specific DNA methylation has been performed on the human nucleosome structure (PDB ID: 1KX5) at the C5 site of every cytosine residue of the DNA sequence (Figure S1) using an in-house Python script. The modified system was subsequently subjected to the full molecular dynamics protocol, including energy minimization and equilibration under NVT and NPT conditions. To ensure sufficient structural relaxation following methylation, the production run was extended from 10 to 100 ns.

### Generation of New Histone Protein Structures of Different Species

There is a lack of nucleosome crystal structures of diverse species in the protein data bank. The amino acid sequence of the histones is available on UniProt (Table S3). The sequences were aligned on UniProt to assess the sequence similarity among the species. The core histones were then folded using Alphafold3.^37^

## Results and Discussion

### Histone Contacts Influence DNA Hydropathy and Hydration in the Nucleosome

The PARCH analysis confirms that DNA is intrinsically hydrophilic, a property dominated by the sugar–phosphate backbone. The negatively charged phosphate groups interact strongly with the surrounding water molecules, forming a robust hydration shell. In contrast, the nucleobases are comparatively hydrophobic and are largely buried within the interior of the double helix through base-stacking interactions (Figure 2). Consistent with our previous work on isolated DNA fragments, we observe a clear quantitative distinction between nucleobase and backbone hydropathy,^28^ with the backbone exhibiting PARCH values that are approximately an order of magnitude more hydrophilic than those of the bases.

**Figure 2:**
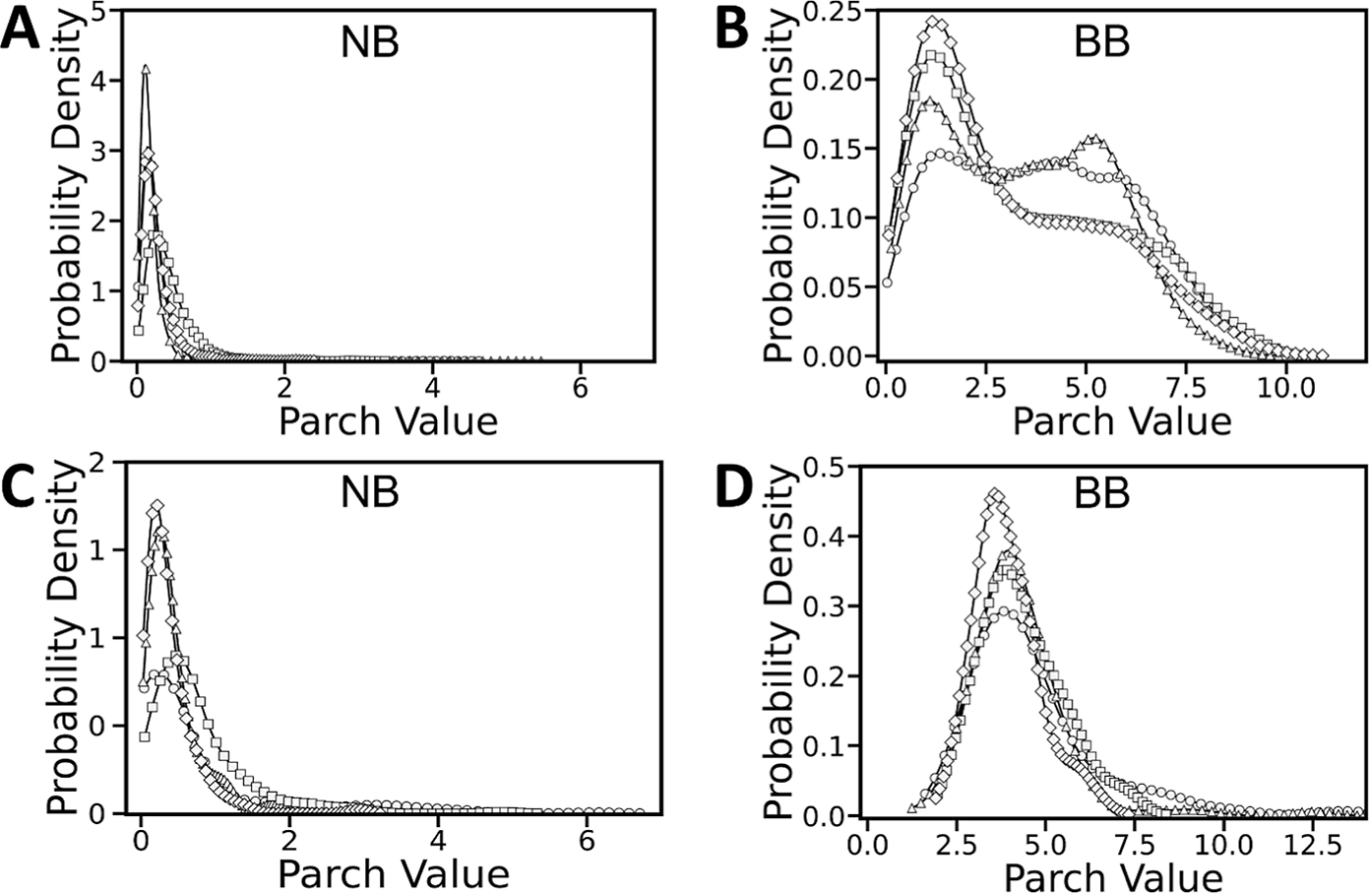
Probability density distributions of PARCH values for DNA for nucleosomes and isolated fragments. (A,B) Distributions for nucleobases (NB) and backbone (BB) in nucleosomal DNA, and the corresponding distributions for DNA fragments (C,D) reported in our previous work (reproduced from ref 28, Copyright 2025, American Chemical Society). Data are shown separately for the four DNA bases: dT (circle, ○), dC (triangle, Δ), dG (square, □), and dA (diamond, ◊). The nucleobase PARCH distributions exhibit qualitatively similar hydrophobic behavior in both nucleosomal DNA and isolated DNA fragments. In contrast, the backbone distributions display a pronounced bimodal character in nucleosomal DNA, indicative of distinct backbone environments arising from histone association and DNA wrapping, compared to the largely unimodal behavior observed for the DNA fragments.

Comparison of nucleosomal DNA with isolated DNA fragments reveals that this intrinsic division between bases and backbone is largely preserved but with important differences arising from nucleosome formation. While nucleobase PARCH values remain essentially unchanged upon nucleosome formation (Figure 2A,C), with consistently low values of approximately 0.3–0.4 for dA, dC, and dT and dG, the DNA backbone exhibits pronounced environment-dependent behavior. In nucleosomal DNA, the backbone PARCH distribution is distinctly bimodal, with prominent peaks near ∼1 and ∼5 (Figure 2B). In contrast, isolated DNA segments display a unimodal backbone PARCH distribution centered near ∼4 (Figure 2D).

To elucidate the origin of the observed bimodality in the backbone PARCH distributions, DNA residues within the nucleosome were classified based on their proximity to histone residues. DNA residues located within 3 Å of any protein atom were designated as proximal, whereas all remaining residues were classified as distal (Figure 3A). Using this criterion, the nucleobases exhibit no statistically significant difference in their PARCH distributions between proximal and distal regions (Figure 3B), consistent with their predominantly buried and stacking-stabilized nature.

**Figure 3:**
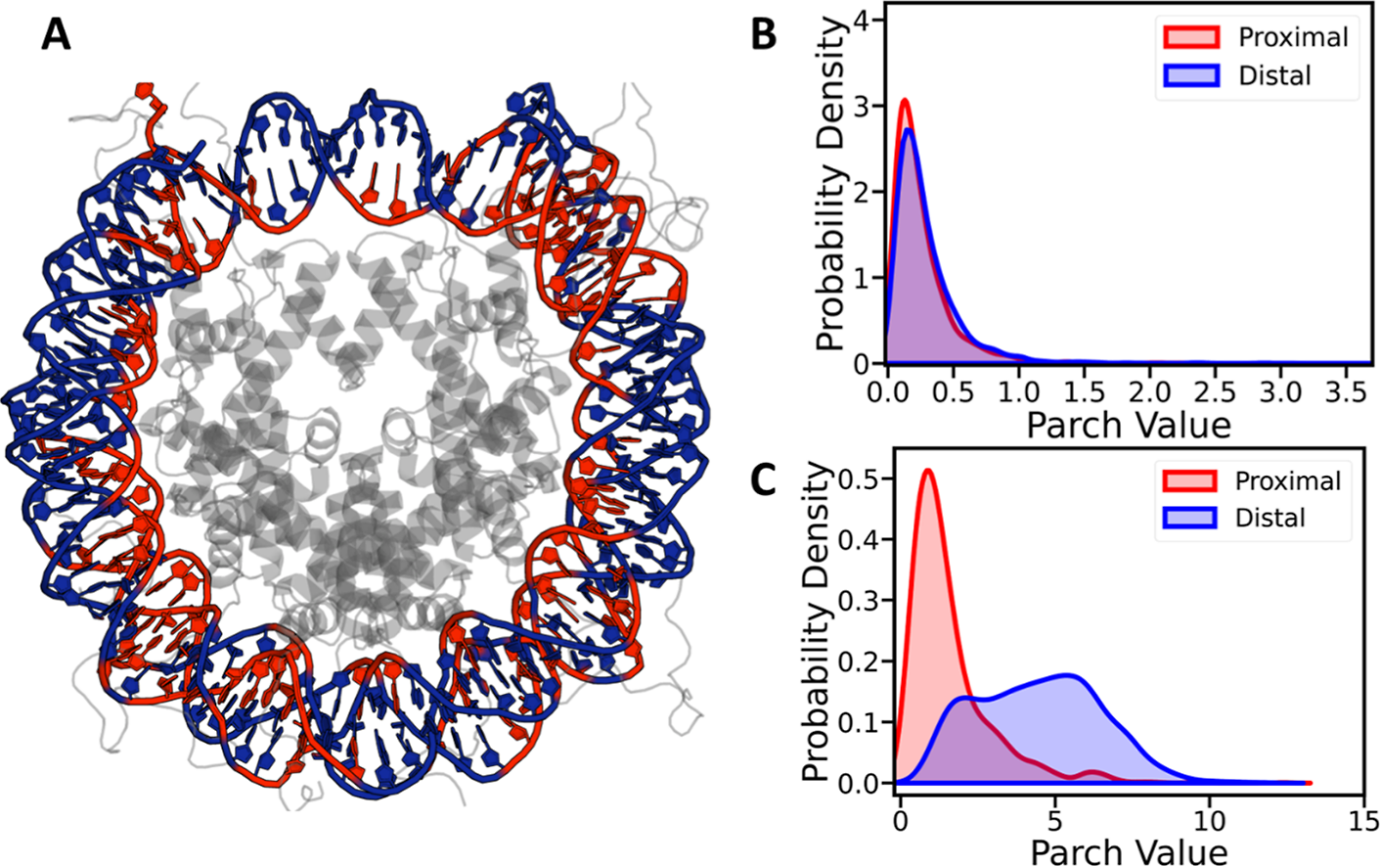
Probability density distributions of PARCH values in nucleosomal DNA relative to the histone core. (A) Representative nucleosome structure showing DNA regions classified as proximal (red) and distal (blue) with respect to the histone core (gray). Probability density distributions of PARCH values for the DNA (B) nucleobases (NB) and (C) backbone (BB). The NB distributions are narrowly peaked, indicating relatively uniform environments, whereas the BB distributions exhibit a pronounced bimodal character, reflecting distinct backbone physicochemical environments arising from histone core–DNA interactions.

In contrast, the DNA backbone displays pronounced environment-dependent behavior. Backbone residues in proximal regions exhibit a broader range of PARCH values spanning approximately 0–6, reflecting increased hydrophobic character associated with direct histone contacts and partial shielding from solvent (Figure 3C). Distal backbone residues, which are more exposed to the aqueous environment, span a wider PARCH range of approximately 1–12 and display predominantly hydrophilic behavior, consistent with enhanced solvent accessibility.

The enhanced hydrophilicity observed for distal backbone residues is consistent with well-established features of DNA hydration.^7^ In free DNA fragments, extensive and ordered water networks have been reported around the sugar–phosphate backbone, contributing to backbone stabilization and strong interactions with the aqueous environment.^38^ Such hydration motifs are expected to be preserved in solvent-exposed regions of nucleosomal DNA, further supporting the interpretation that histone proximity selectively modulates backbone hydropathy without substantially altering the nucleobase behavior.

Taken together, our PARCH results reflect that histone proximity significantly reshapes the hydropathy landscape of the nucleosomal DNA backbone. Histone association locally diminishes backbone hydrophilicity by replacing water interactions with protein–DNA contacts and structured interfacial waters, while distal regions away from the histone octamer retain conventional hydration signatures of free DNA. This differential hydropathy has implications for nucleosome stability, dynamics, and accessibility and reinforces structural models in which tight DNA wrapping is facilitated by specific histone interactions that reshape local DNA solvation.

### PARCH Data Confirms the Role of Arginine Anchors in Nucleosomal DNA

We examined a well-defined set of highly conserved arginine residues that constitute a hallmark feature of histone–DNA interactions in nucleosomes (Figure S2). These arginine residues insert into the DNA minor groove and act as structural anchors that stabilize DNA wrapping around the histone octamer. High-resolution X-ray crystal structures of the nucleosome core particle show that these residues are positioned at regularly spaced intervals along the histone surface, coinciding with locations where the DNA minor groove faces inward toward the histone core.^4,5,39–56^ At these sites, the DNA undergoes sharp bending and local compression, deformations that would be energetically unfavorable without stabilizing protein contacts.^4,5,39–56^

To quantify the contribution of these arginine–DNA interactions, we computed PARCH values for histone proteins under two conditions: (i) within the intact nucleosome and (ii) in the absence of DNA, generated by removing the DNA from the complex. Comparing PARCH values between these two states provides direct insight into how DNA binding alters the local physicochemical environment of histone residues, particularly at anchoring sites.

Across all 13 nucleosome systems analyzed, the arginine anchor residues exhibit consistently lower PARCH values in the presence of DNA (Table 1). At first glance, this result may appear counterintuitive given the highly charged and hydrophilic nature of DNA. However, the flexible arginine side chain and planar guanidinium group are uniquely suited for insertion into the minor groove, where they form multiple, simultaneous hydrogen bonds while maintaining strong electrostatic interactions with the negatively charged phosphate backbone. These close, multivalent contacts effectively reduce the local solvent exposure, leading to lower PARCH values for arginine residues engaged in anchoring interactions. In most cases, arginine side chains interact with both phosphate oxygens and base-edge atoms, stabilizing DNA positioning without disrupting base pairing. Notably, these interactions are largely sequence independent, consistent with the role of DNA shape and flexibility rather than base-specific recognition in nucleosome formation.

**Table 1:**
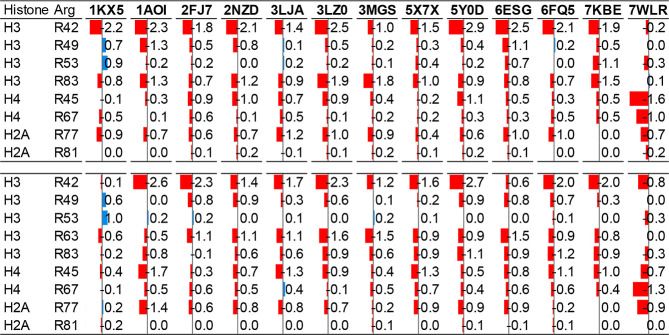
PARCH Interpretation of Conserved Arginine Anchors at Histone–DNA Contact Sites^a^ ^,^ ^b^ ^,^ ^c^

a Shown are ΔPARCH values for conserved arginine residues across histone cores, comparing values calculated in the intact nucleosome and in the corresponding histone-only systems (DNA present–DNA removed). Color intensity reflects the magnitude and sign of the ΔPARCH value (blue, more hydrophilic; red, reduced hydrophilicity).

b Arginine residues that insert into the DNA minor groove exhibit systematically reduced local hydrophilicity associated with tight minor-groove insertion and multivalent protein–DNA contacts.

c This trend is observed consistently across 13 nucleosomes studied here, highlighting the role of conserved arginine anchors in modulating the physicochemical environment at histone–DNA interfaces.

Structural analyses identify a conserved set of arginine anchors across histones H3, H4, and H2A (Figure S2), and these residues are preserved across eukaryotic species, underscoring their functional importance. Experimental mutation or chemical modification of these arginine residues weakens histone–DNA binding, reduces nucleosome stability, and increases DNA unwrapping. Beyond their static structural role, arginine anchors also contribute to nucleosome dynamics. Molecular dynamics simulations and experimental studies indicate that arginine–minor groove contacts can transiently break and reform, enabling local DNA breathing while maintaining overall nucleosome integrity. This balance between stability and flexibility is essential for transcription factor binding, chromatin remodeling, and nucleosome sliding.

To place the arginine anchors in a broader context, we also analyzed other histone contact residues, defined as any protein residue whose center of geometry lies within 8 Å of the center of geometry of any DNA residue (Figure 4). While many contact residues show detectable hydropathy changes upon DNA binding, the magnitude and consistency of the shifts are strongest for the conserved arginine anchors. This distinction highlights the unique role of arginine residues as primary physicochemical mediators of histone-DNA anchoring rather than generic electrostatic contributors.

**Figure 4:**
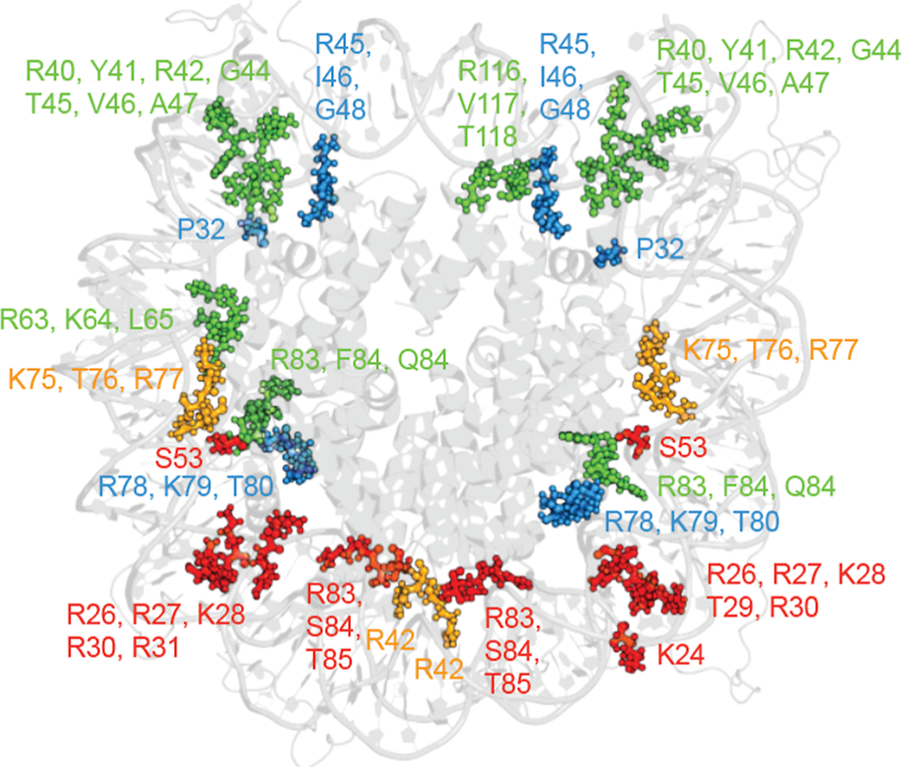
Histone residues contacting DNA in the nucleosome. Shown are the DNA-contacting histone residues in the nucleosome core particle (PDB ID: 1KX5). Amino acid residues were identified based on their proximity to DNA within an 8 Å cutoff. The histone core and the DNA are shown in gray for context, while contacting residues are color-coded by histone type: H3 (green), H4 (cyan), H2A (yellow), and H2B (red).

Together, these results demonstrate that conserved arginine insertions into the DNA minor groove represent a fundamental design principle of nucleosome organization. By combining strong electrostatic interactions with localized reductions in hydrophilicity, these anchors stabilize the extreme DNA curvature required for chromatin compaction while preserving the dynamic accessibility essential for genome regulation.

### Acidic Patch Hydropathy on Histones Is Largely Insensitive to DNA Wrapping

The acidic patch is a highly conserved, negatively charged surface region of the nucleosome formed by acidic residues on histones H2A (E56, E61, E64, D90, E91, and E92) and H2B (E102 and E110). Each nucleosome contains two acidic patches, one on each face of the histone octamer. Unlike the DNA-binding interface, the acidic patch functions primarily as a protein–protein interaction hub, mediating nucleosome–nucleosome contacts and serving as a docking site for histone tails, chromatin remodelers, histone chaperones, and viral proteins, often through arginine-rich motifs that exploit electrostatic complementarity.

Comparison of the hydropathy of acidic patch residues in the presence and absence of wrapped DNA reveals that DNA association has a minimal impact on the hydropathy of most acidic patch residues (Table 2). This behavior is consistent with the largely protein-exposed nature of the acidic patch and its limited direct interaction with DNA, reinforcing its role as a stable interaction surface rather than a DNA-contacting interface.

**Table 2:**
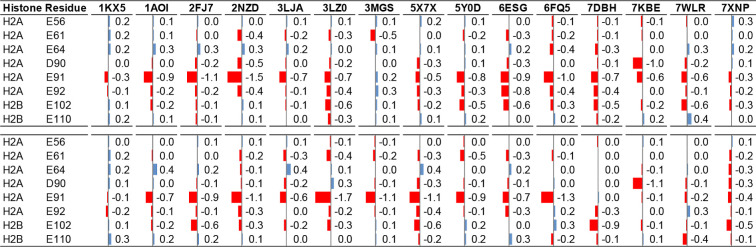
PARCH Analysis of Acidic Patch Residues across Nucleosome Systems^a^ ^,^ ^b^ ^,^ ^c^ ^,^ ^d^

a Shown are ΔPARCH values for acidic patch residues on histones H2A (E56, E61, E64, D90, E91, and E92) and H2B (E102 and E110) across multiple nucleosome structures.

b ΔPARCH values were calculated as the difference between the intact nucleosome and the corresponding histone-only system (DNA present—DNA removed).

c Color intensity reflects the magnitude and sign of the ΔPARCH value (blue, more hydrophilic; red, reduced hydrophilicity).

d Across all systems, DNA wrapping around histones has minimal impact on the hydropathy of most acidic patch residues; notably, H2A E91 exhibits a consistent decrease in PARCH value in a nucleosome

Notably, H2A E91 exhibits a reproducible decrease in PARCH values upon nucleosome assembly, indicating a localized change in its physicochemical environment. This trend is observed consistently across all 13 nucleosome systems analyzed, highlighting the overall hydropathy stability of the acidic patch while identifying E91 as a subtle but systematic exception. Importantly, H2A E91 has been repeatedly identified in the literature as a core and a functionally significant component of the acidic patch. Structural and biochemical studies place E91 near key arginine-binding pockets used by chromatin remodelers, histone tails, and viral proteins, and mutational and cross-linking analyses implicate this residue in acidic patch-dependent binding and regulation. Computational and structural investigations further suggest that E91 can participate in local salt-bridge networks and conformational coupling within the acidic patch, supporting its role in stabilizing an interaction-ready geometry. In this context, the PARCH decrease observed for E91 upon nucleosome assembly is consistent with prior work that identified this residue as a particularly sensitive and functionally engaged element of the acidic patch microenvironment.

### Core Histone Hydropathy Is Conserved across Species

To assess whether histone physicochemical properties vary across species, we examined the hydropathy of core histone domains, focusing exclusively on the folded histone cores and excluding the N- and C-terminal tails for clarity. Histone cores are known to be highly conserved in sequence and structure across eukaryotes, forming the stable scaffold of the nucleosome, whereas tails are more variable and primarily involved in regulatory interactions.^4,10^ Due to the limited availability of high-resolution crystal structures for some organisms, we analyzed histones from eight species using a combination of experimentally resolved and predicted structures. Specifically, crystal structures were available for *Komagataella pastoris*,^52^ *S. cerevisiae*,^57^ *D. melanogaster*, *G. gallus*,^14^ *M. musculus*,^50^ and *H. sapiens*,^5^ while histone core structures for *H. vulgaris* and *S. scrofa* were generated using AlphaFold3 ^37^ predictions based on UniProt sequences (Table S3).

PARCH analysis of the core histone residues reveals no statistically significant differences in hydropathy distributions across the eight species, indicating that histone core hydropathy is strongly conserved despite evolutionary divergence (Figures S3,S4). This result is consistent with prior structural and comparative studies showing that the histone fold domains maintain nearly identical three-dimensional architectures and interaction patterns with DNA across eukaryotes.^4,10^ The conservation of PARCH profiles suggests that beyond sequence and structure the hydropathy landscape of the histone core is an evolutionarily constrained property, likely reflecting the need to preserve stable histone–DNA interactions and nucleosome integrity across species. These findings further support the view that species-specific chromatin behavior arises primarily from differences in histone tails, variants, and post-translational modifications rather than from alterations in the core histone physicochemical framework.^15,58^

#### Dinucleosome Formation Reduces Inner DNA Backbone Hydrophilicity

Dinucleosomes represent the simplest higher-order chromatin assembly, consisting of two nucleosomes connected by linker DNA.^10,12,13^ They serve as a critical intermediate between isolated nucleosomes and folded chromatin fibers and are central to understanding how local nucleosome–nucleosome interactions shape chromatin organization.^12,18^ Structural and biochemical studies show that dinucleosomes adopt multiple relative orientations, stabilized primarily through protein–protein interactions involving histone tails and the acidic patch, as well as through bending and positioning of the linker DNA.^18^

High-resolution cryo-EM and biochemical analyses demonstrate that dinucleosome formation introduces spatial asymmetry, creating regions that are buried at the nucleosome–nucleosome interface and regions that remain solvent-exposed.^12^ Inward-facing DNA segments, histone tail contact regions, and acidic patch-H4 tail interfaces experience reduced solvent accessibility, while outward-facing DNA and histone surfaces remain highly hydrated.

Our PARCH analysis provides a quantitative physicochemical interpretation of this organization. For the dinucleosome analysis, the classification of residues as inner or outer was defined based on internucleosome proximity rather than distance from the interior of a single nucleosome. Specifically, residues from one nucleosome were classified as inner if any protein or DNA atom was within 20 Å of any protein or DNA atom from the adjacent nucleosome. This criterion identifies residues located at or near the nucleosome–nucleosome interface in the dinucleosome assembly. Because this study examines a single experimentally resolved dinucleosome configuration, we did not systematically vary the distance threshold. However, we note that the appropriate cutoff may depend on the relative orientation and compaction state of the dinucleosome, such as open, closed, or intermediate conformations. Future analyses across multiple dinucleosome geometries will allow a further assessment of the robustness of this criterion.

Based on the 20 Å cutoff, DNA backbone residues exhibit significantly lower PARCH values in inner, histone-facing regions compared to outer, solvent-facing regions, indicating reduced hydrophilicity at buried interfaces (Figure 5). This effect is most pronounced for the DNA backbone, modest for nucleobases, and negligible for histone residues. In the context of dinucleosomes, this suggests that internucleosomal interfaces preferentially sequester DNA backbone segments, while histone surfaces largely retain their hydration properties. Such selective modulation of DNA hydropathy provides a mechanism for stabilizing dinucleosome assemblies without requiring large changes in histone chemistry.

**Figure 5:**
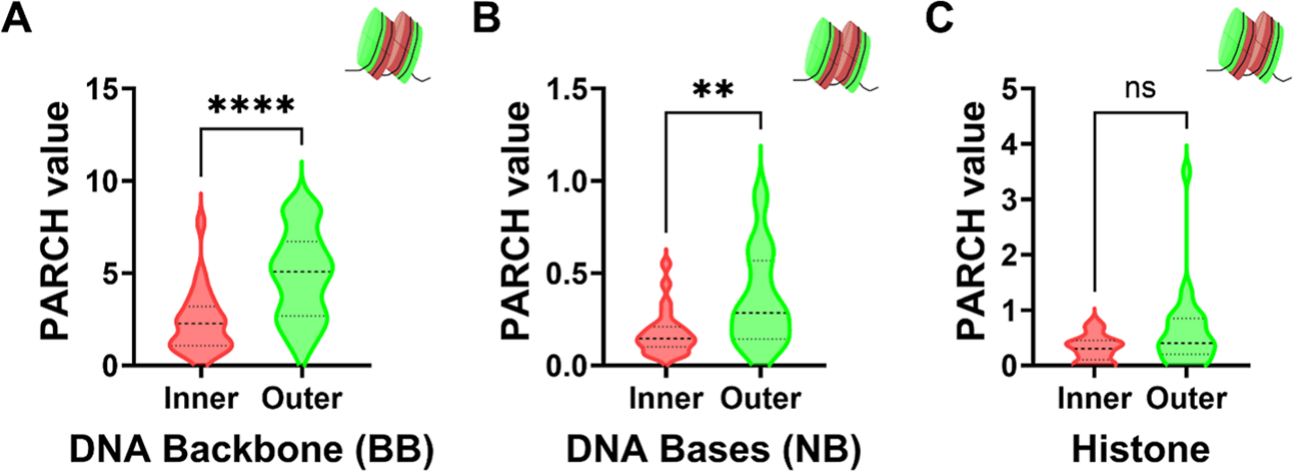
Comparison of PARCH values for inner and outer regions of a dinucleosome (PDB ID:3LEL). Violin plots show the distributions of PARCH values for (A) DNA backbone (BB), (B) DNA bases (NB), and (C) histone residues, classified by their spatial location relative to the nucleosome interior (inner, nucleosome-facing; within 20 Å cutoff) and exterior (outer, solvent-facing). The DNA backbone exhibits higher PARCH values in outer regions compared to inner regions, indicating decreased hydrophilicity upon formation of the nucleosome–nucleosome interface. DNA bases show a smaller but statistically significant difference between inner and outer regions, while histone residues display no significant change. Statistical significance is indicated as ****p < 0.0001, **p < 0.01, and ns, not significant.

Importantly, experimental studies show that dinucleosome formation does not dramatically alter nucleosome core structure but instead modulates DNA accessibility, linker geometry, and histone tail interactions.^14,59^ The PARCH trends observed here are fully consistent with this picture: hydropathy changes are localized and interface-specific rather than global. Reduced backbone hydrophilicity at inner regions would favor close DNA packing and reduced electrostatic repulsion at nucleosome–nucleosome interfaces, while preservation of histone hydropathy supports continued dynamic binding of chromatin-associated factors.

These results position hydropathy as an additional quantifiable layer governing dinucleosome stability and chromatin folding. By revealing that chromatin compaction is accompanied by selective redistribution of DNA hydropathy rather than uniform dehydration, PARCH provides a mechanistic bridge between structural observations of dinucleosomes and their dynamic regulatory behavior.

### Cytosine Methylation Reduces DNA Backbone Hydrophilicity in the Nucleosome

Cytosine methylation is one of the most extensively studied DNA modifications and occurs at position 5 of the pyrimidine ring to form 5-methylcytosine (FMC). This epigenetic modification plays a central role in gene regulation and chromatin organization by modulating DNA accessibility without altering the underlying nucleotide sequence.^60–62^ At the molecular level, cytosine methylation introduces additional hydrophobic character into the DNA major groove and alters local DNA shape, flexibility, and hydration, all of which are known to influence DNA–histone interactions and nucleosome stability.^23–25^

To isolate and quantify the physicochemical consequences of cytosine methylation in a nucleosomal context, we constructed a fully methylated nucleosome model by methylating all cytosine residues in the canonical nucleosome structure (PDB ID: 1KX5) using an in-house script (Figure S2). This modeling strategy enables a systematic assessment of methylation-induced hydropathy changes using PARCH, independent of sequence heterogeneity or partial methylation patterns, and provides a controlled framework for evaluating how cytosine methylation reshapes DNA hydropathy within the nucleosome.

PARCH analysis reveals a clear and systematic methylation-induced effect on the DNA backbone. As shown in Figure 6, DNA backbone residues associated with 5-methylcytosine exhibit a statistically significant reduction in PARCH values compared with those associated with unmethylated cytosine, indicating a decreased backbone hydrophilicity upon methylation. Notably, this shift occurs despite the chemical modification being localized to the nucleobase rather than the sugar–phosphate backbone, demonstrating that a small, localized hydrophobic perturbation can propagate through the nucleotide to alter the physicochemical environment of the backbone.

**Figure 6:**
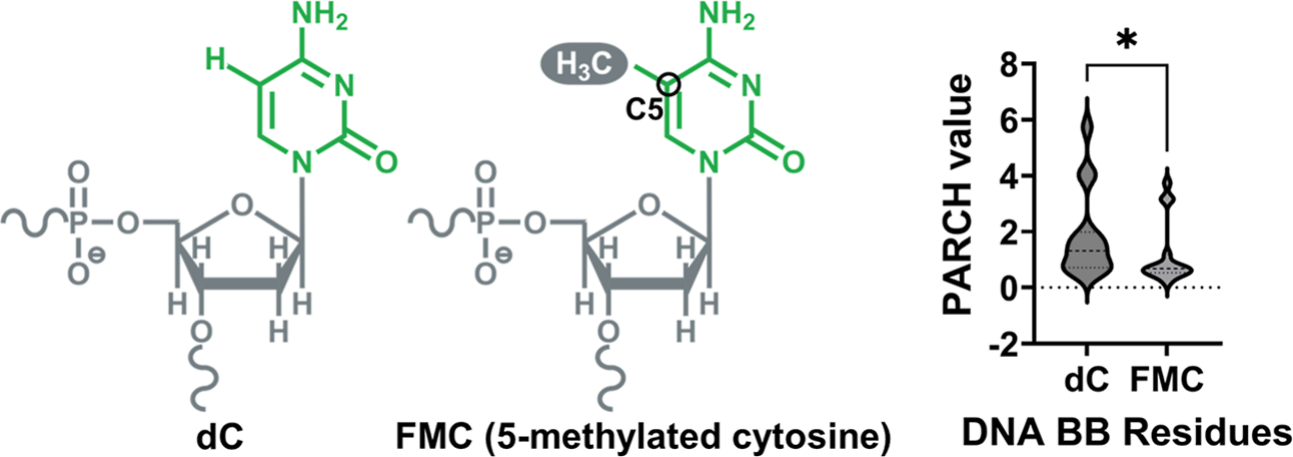
Effect of cytosine methylation on DNA backbone hydropathy quantified by PARCH. Schematic representations of unmethylated cytosine (dC) and 5-methylcytosine (FMC) are shown, highlighting the addition of the methyl group at the 5-position of the pyrimidine ring. Violin plots on the right compare the distributions of PARCH values for DNA backbone (BB) residues associated with dC and FMC. Cytosine methylation leads to a statistically significant decrease in backbone PARCH values, indicating reduced hydrophilicity of the DNA backbone upon methylation. The asterisk denotes statistical significance (*p < 0.05).

Residue-level analysis further confirms this backbone-dominated response. As summarized in Table 3, DNA backbone residues exhibit predominantly negative ΔPARCH values following methylation, whereas nucleobase residues display substantially smaller and more variable ΔPARCH shifts. This contrast indicates that cytosine methylation primarily perturbs backbone hydropathy rather than nucleobase hydropathy, reinforcing the central role of the sugar–phosphate backbone in mediating methylation-dependent changes in DNA–histone interactions.

**Table 3:**
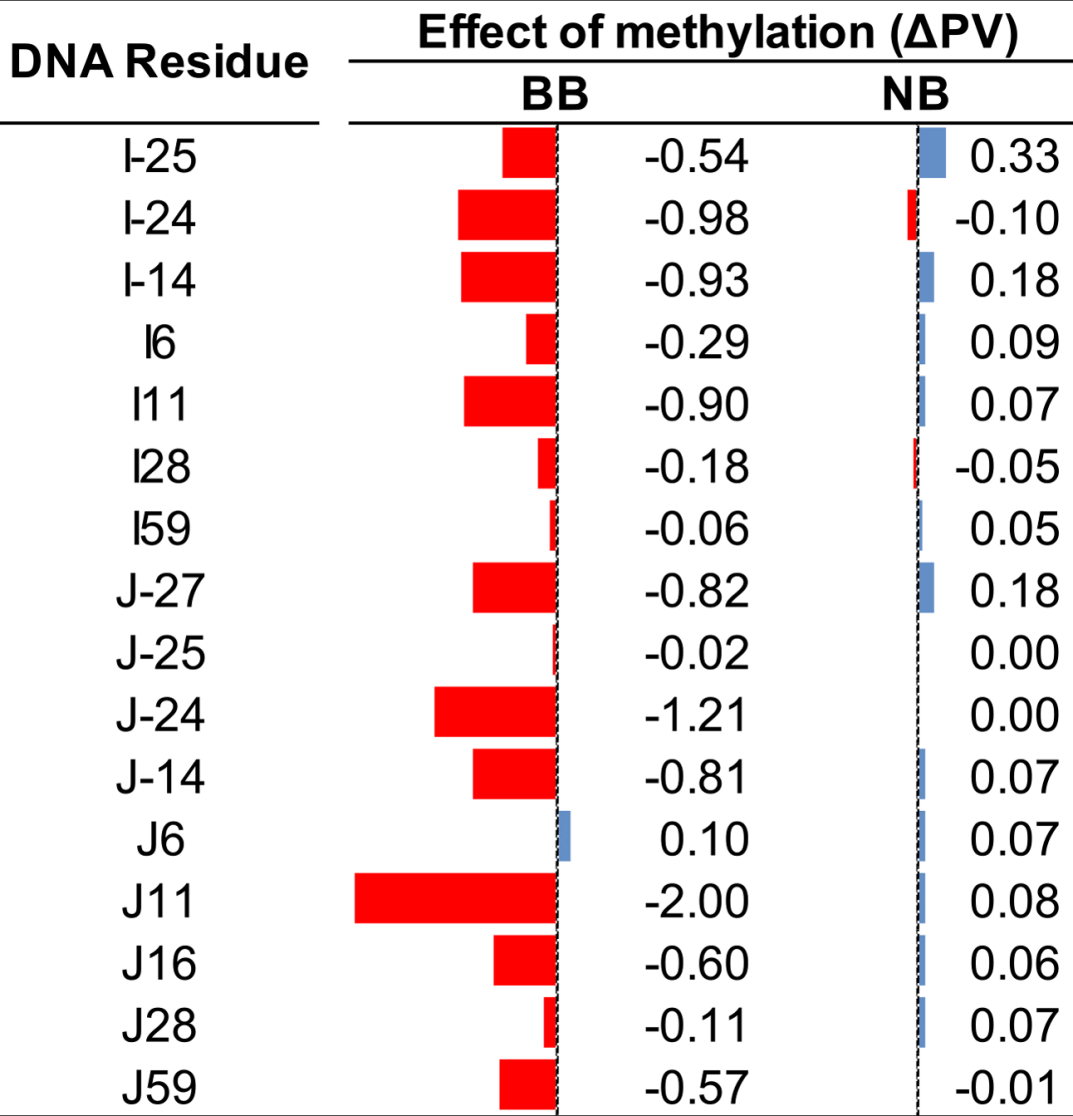
Effect of Cytosine Methylation on DNA Hydropathy Quantified by PARCH (PDB ID: 1KX5)­^a^ ^,^ ^b^ ^,^ ^c^ ^,^ [Table-fn t2fn4]

a Shown are the changes in PARCH values (ΔPV) upon cytosine methylation for DNA backbone (BB) and nucleobases (NB) for residues that make contacts with histones (8 Å cutoff).

b ΔPV values were calculated as the difference between methylated and unmethylated DNA states.

c Negative ΔPV values (red) indicate reduced hydrophilicity upon methylation, while positive values (blue) indicate increased hydrophilicity.

These results are consistent with prior experimental and computational studies showing that cytosine methylation enhances nucleosome stability and suppresses DNA breathing by modifying DNA flexibility and hydration, rather than inducing large-scale structural changes in the nucleosome core.^23–25^ The PARCH results provide a quantitative physicochemical basis for these observations by directly showing that methylation reduces backbone hydrophilicity, which would favor tighter DNA–histone association and decreased solvent exposure at histone-facing regions.

Importantly, these methylation-induced PARCH trends align with our broader analyses of nucleosomes, where the reduced backbone hydrophilicity correlates with buried or protein-contacting DNA regions. In this context, cytosine methylation appears to reinforce nucleosome stability by selectively shifting DNA backbone hydropathy toward a less hydrated state without requiring significant changes in the hydropathy of histones or nucleosome core structure. This interpretation is consistent with genome-wide studies reporting increased nucleosome occupancy and positioning stability in methylated regions.^23–25,60–62^ These results identify DNA backbone hydropathy as a key physicochemical mediator, through which cytosine methylation contributes to chromatin compaction and epigenetic regulation.

## Conclusions

In summary, this work establishes hydropathy as a central, quantifiable physicochemical principle underlying chromatin organization. Using PARCH, we show that histone association selectively reshapes the hydropathy landscape of DNA, primarily by modulating the sugar–phosphate backbone while leaving nucleobase hydropathy largely unchanged. Conserved arginine anchors emerge as key determinants that locally reduce backbone hydrophilicity at histone–DNA contact sites, stabilizing extreme DNA bending without compromising dynamic accessibility. In contrast, the nucleosomal acidic patch remains hydropathy-stable upon DNA wrapping, consistent with its role as a protein–protein interaction hub. Across species, histone core hydropathy is strikingly conserved, underscoring strong evolutionary constraints on the physicochemical properties of the nucleosome scaffold. Extension to dinucleosomes reveals that higher-order chromatin assembly is accompanied by selective reduction of DNA backbone hydrophilicity at buried interfaces rather than global dehydration or changes in histone chemistry. Finally, cytosine methylation is shown to selectively decrease the DNA backbone hydrophilicity, providing a quantitative physicochemical mechanism for enhanced nucleosome stability and chromatin compaction. Together, these results position PARCH as a robust framework for linking structural, biochemical, and epigenetic features of chromatin to their underlying hydropathy landscapes, offering new insight into how genome packaging and regulation emerge from localized, environment-dependent modulation of DNA–protein interactions.

## Supplementary Material



## Data Availability

All computed PARCH scale values for the data set is available at https://github.com/NangiaLab/PARCHValuesNucleosomes/. There is no restriction on the use of the data.

## Notes

List of PDB IDs of nucleosome structures, PARCH simulation parameters, Uniport IDs of histones, methylation of DNA cytosine residues, protein sequence alignment histone of eight species, PARCH analysis of histones, and statistical analysis of PARCH values of eight different histones (PDF)
