# Supplementary material for: Hydropathy Landscapes of Histone–DNA Interactions in Chromatin Building Blocks
Source: J Phys Chem B. 2026 Mar 27;130(14):3844–54. doi: 10.1021/acs.jpcb.6c00520 (PMC13071913; doi:10.1021/acs.jpcb.6c00520)
Supplement: Supplementary file 1 [file jp6c00520_si_001.pdf]

Supporting Information

Hydropathy Landscapes of Histone–DNA Interactions in Chromatin Building Blocks

Ratnakshi Mandal, Andre Christophe Noel, Anna M Baur and Shikha Nangia*

Department of Biomedical and Chemical Engineering, Syracuse University, Syracuse, NY 13244, USA

Contents

Table S1: List and short description of nucleosome structures. 3
Table S2: Parameters used for PARCH calculations of nucleosomes 4
Table S3: List of UniProt IDs used to make histone structures. 5
Figure S1. Methylation of DNA dC residues... 6
Figure S2. Histones of eight different species aligned 7
Figure S3. PARCH analysis of histones of eight different histones... 8
Figure S4. Statistical analysis of the PARCH values of the eight different histones................. 9
References……………………………………………………………………………………………10

Table S1: List and short description of nucleosome structures.

PDB ID	Description
1KX5[1]	X Ray structure of Nucleosome at 1.9 Å resolution
1AOI[2]	Nucleosome complex between histones and 146 long base pairs of DNA
2FJ7[3]	Nucleosome with poly (dA dT) sequence
2NZD[4]	Nucleosome with 145 base pairs of DNA
3LJA[5]	Nucleosome image with divalent metal binding
3LZ0[6]	Nucleosome with 601 Widom DNA sequence
3MGS[7]	Nucleosome with Cesium ions binding
3WKJ[8]	Nucleosome with human TSH2B
5B31[9]	Crystal structure of the heterotypic H2AZ/H2A nucleosome with H3.1
5X7X[10]	Nucleosome containing H3.3 at 2.18 Å
5Y0D[11]	Human nucleosome containing the H2B E76K mutant
6ESG[12]	Nucleosome breathing: Class 2
6FQ5[13]	Class 1 canonical nucleosome
7DBH[14]	Mouse nucleosome structure
7KBE[15]	Nucleosome isolated from metaphase chromosome
7XNP[16]	Nucleosome AAG complex
7WLR[17]	Nucleosome with *Komagataella pastoris* histones
8ETT[18]	Class 1 of the INO80-Hexasome complex
7YOZ[19]	Human sub-nucleosome
1ID3[20]	Yeast nucleosome
2NQB	*Drosophila* nucleosome structure (https://www.rcsb.org/structure/2NQB)
4QLC[21]	Chromatosome at 3.5 Å
3LEL[22]	Structural insight into the sequence dependence of nucleosome positioning

Table S2: Parameters used for PARCH calculations of nucleosomes

Parameter	Description	Protein	Nucleic Acids
d_{max}	Distance between the center of geometry of the molecule to the farthest point on the protein surface	Depends on size of molecule	Depends on size of molecule
$d_{interion}$	Maximum distance between any two counterions	3.0 nm	3.0 Å
d_{ion}	Radial distance between surface of molecule and counterions	3.0 nm	Minimum 3.0 Å, smallest distance that accommodates all counter-ions with $d_{interion}$.
d_b	Distance between counterion to box boundary	3.0 nm	3.0 Å
l	Length of simulation box	$2\times(d_{max}+d_{ion}+d_b)$	$2\times(d_{max}+d_{ion}+d_b)$
d_{shell}	Water layer thickness from the surface of the molecule, when solvated	4.15 Å	4.5 Å
d_{water}	Cut-off used to calculate the number of water molecules around each residue	3.15 Å	4.5 Å

Table S3: List of UniProt IDs used to make histone structures.

UniProt ID	Description
A0A1V0IG74	*Hydra vulgaris* histone H2A
A0A1V0IGH5	*Hydra vulgaris* histone H2B
T2MEU0	*Hydra vulgaris* histone H3
A0A8B6XYN7	*Hydra vulgaris* histone H4
A0A4X1SGV6	*Sus scrofa* histone H2A
A0A4X1VFY8	*Sus scrofa* histone H2B
K9IWF3	*Sus scrofa* histone H3
P62802	*Sus scrofa* histone H4

Figure S1. Methylation of DNA dC residues

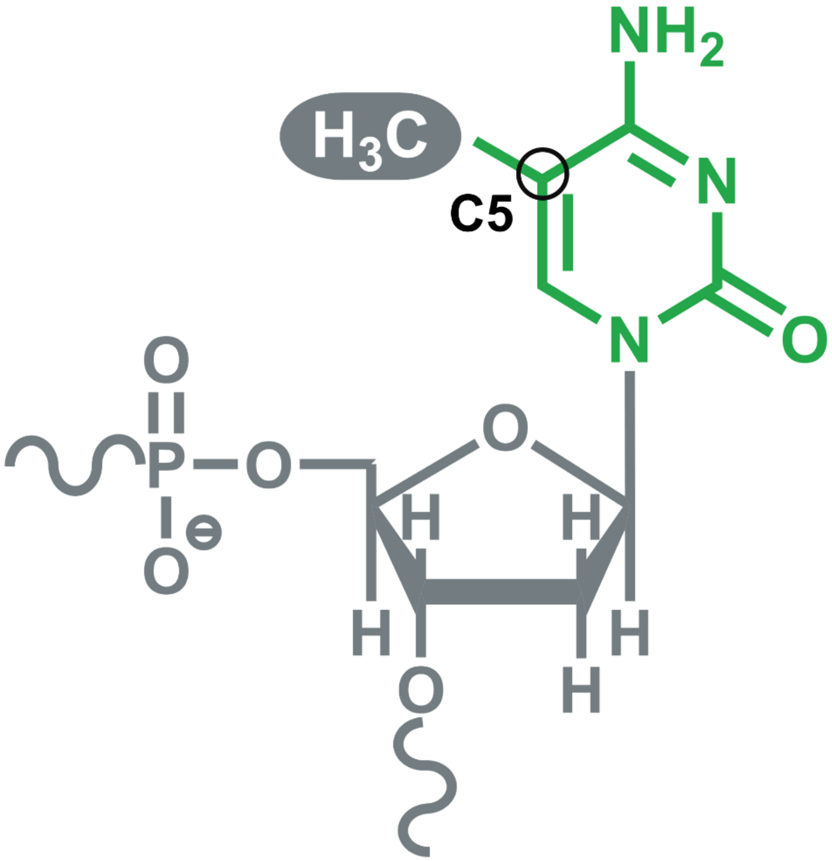

FMC (5-methylated cytosine)

Site-specific DNA methylation has been performed on the human nucleosome structure (PDB ID: 1KX5) at the C5 site of every cytosine residue of the DNA sequence using an in-house Python script. The script parses the Protein Data Bank (PDB) file to identify cytosine (dC) residues and their associated H5 hydrogen atom and replaces it with a methyl (CH3) group. The H5 atom is replaced by a C5M carbon atom, and additional hydrogen atoms (H51, H52, and H53) are added to the PDB file. The atomic positions of the H51, H52, and H53 atoms are calculated assuming sp3 hybridization, with bond lengths of C5M-H5x = 1.09 Å and bond angles of 109.5°. The methyl group is positioned along the vector from the adjacent carbon, C5, using normalized direction vectors. The positions of 2 H5x atoms were determined by vector rotation around the z axis to avoid steric clashes. The structure was equilibrated for 200 ns to relax bonds and optimize the methylated residue.

The ITP files of the DNA strands were also modified to be compatible with the CHARMM general forcefield (CGenFF) for the base atoms and CHARMM36 for the backbone atoms.

Figure S2. Histones of eight different species aligned

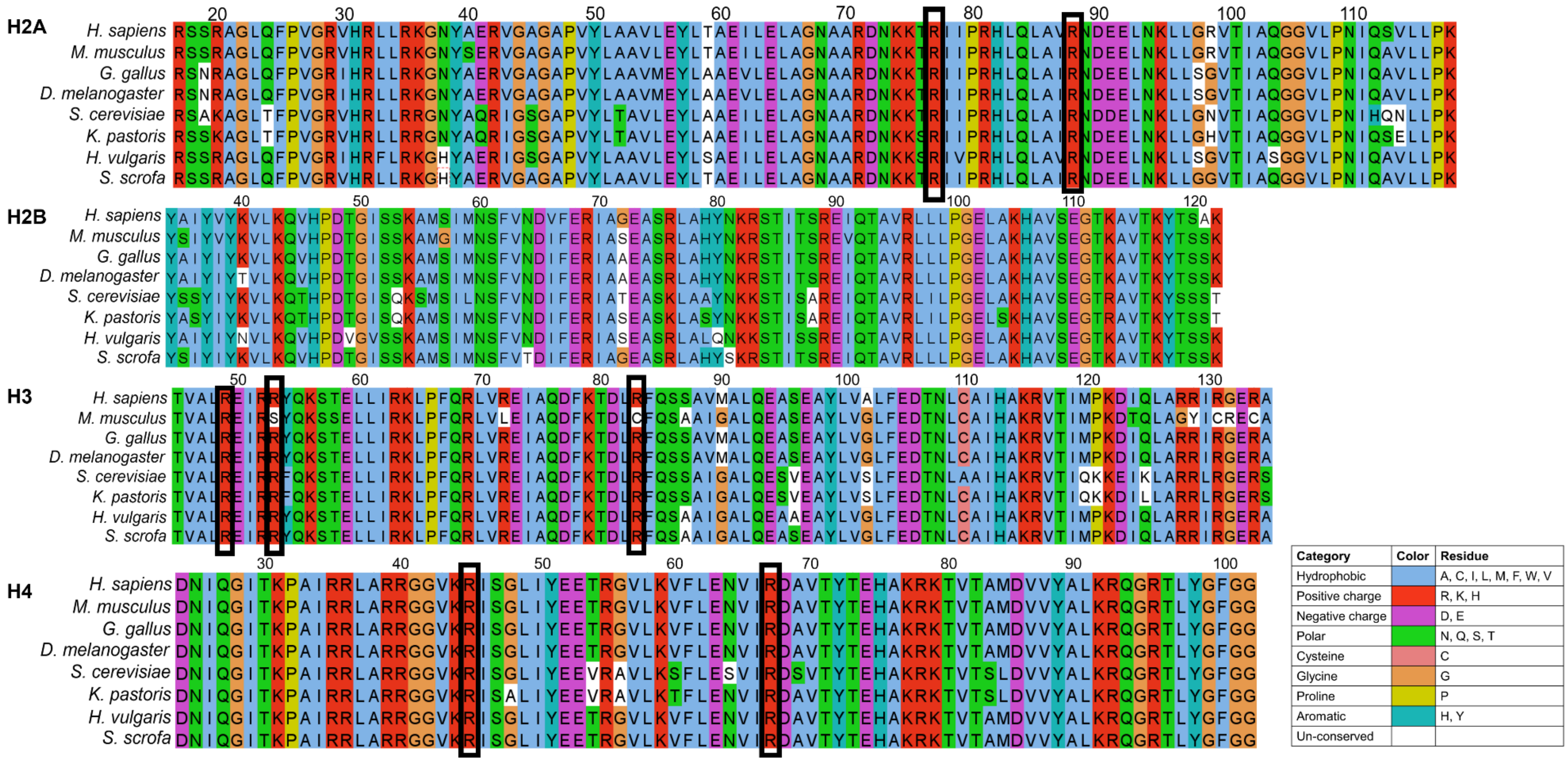

Category	Color	Residue
Hydrophobic		A, C, I, L, M, F, W, V
Positive charge		R, K, H
Negative charge		D, E
Polar		N, Q, S, T
Cysteine		C
Glycine		G
Proline		P
Aromatic		H, Y
Un-conserved		

The conserved arginine anchors are highlighted in the histones across eight species. H3 of *M. musculus* shows different amino acids in positions 53 and 83, as the structure contains an H318mm variant.

Figure S3. PARCH analysis of histones of eight different histones

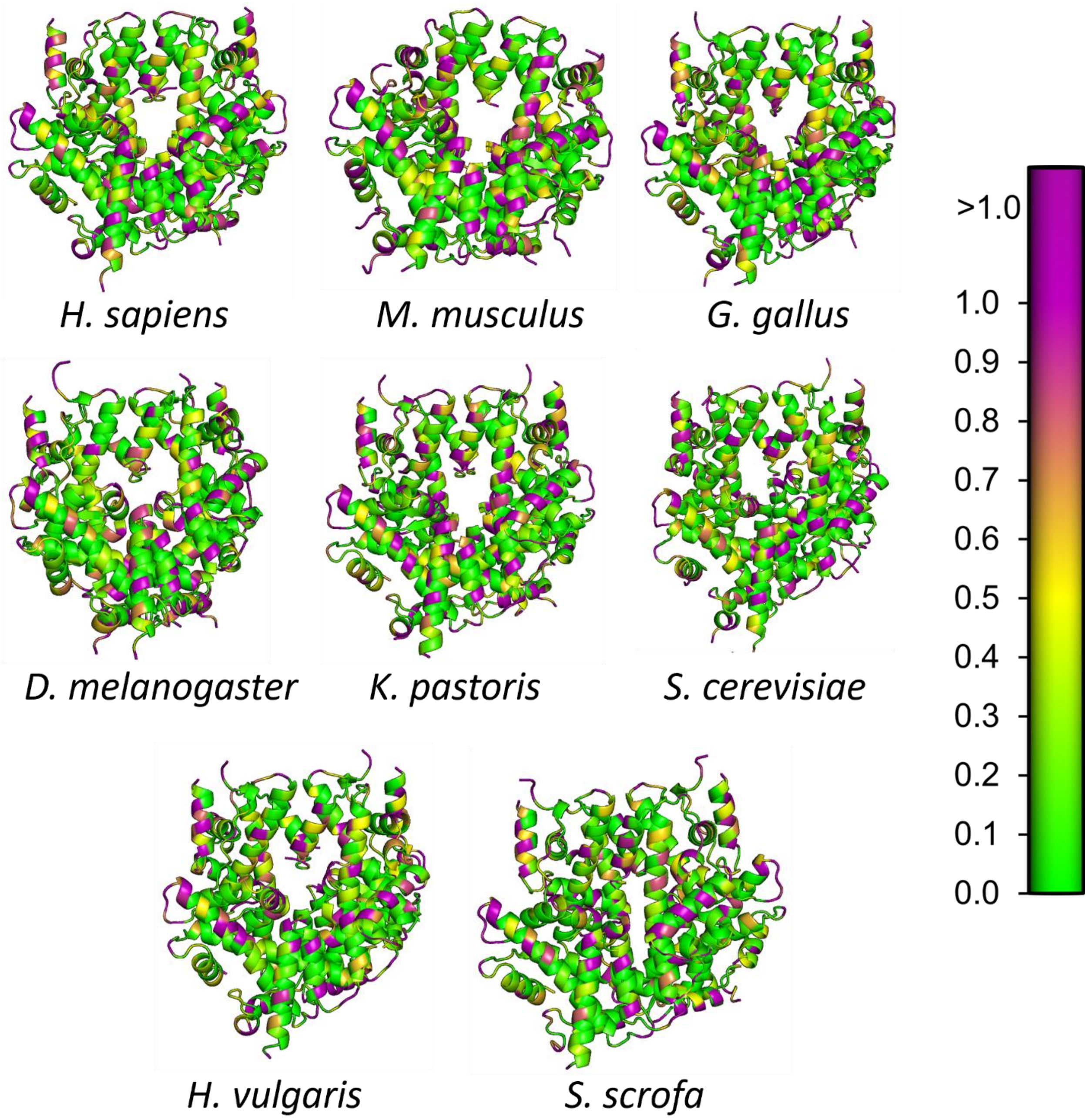

Figure S4. Statistical analysis of the PARCH values of the eight different histones

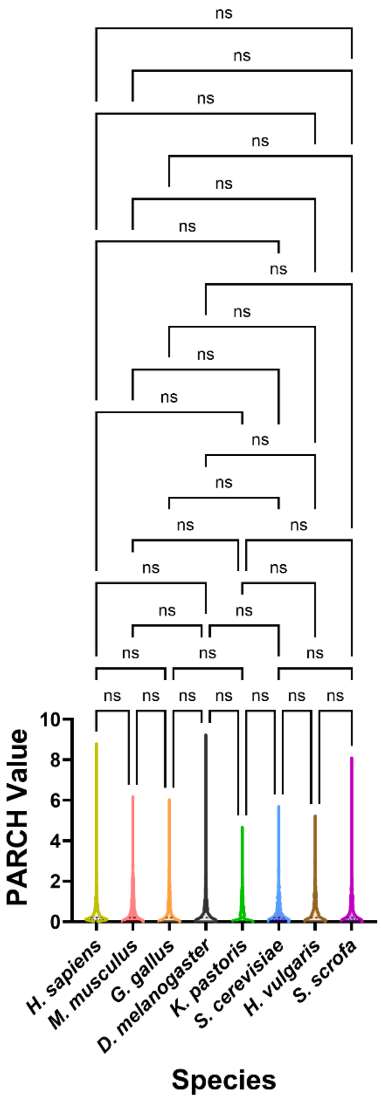

The statistical analysis shows that the PARCH values of the histones of 8 different species are not significantly (ns) different from each other, as determined by the ANOVA test (p=0.2920).

References

(1) Davey, C. A.; Sargent, D. F.; Luger, K.; Maeder, A. W.; Richmond, T. J. Solvent Mediated Interactions in the Structure of the Nucleosome Core Particle at 1.9Å Resolution††We dedicate this paper to the memory of Max Perutz who was particularly inspirational and supportive to T.J.R. in the early stages of this study. *Journal of Molecular Biology* **2002**, *319* (5), 1097–1113. DOI: https://doi.org/10.1016/S0022-2836(02)00386-8.

(2) Luger, K.; Mäder, A. W.; Richmond, R. K.; Sargent, D. F.; Richmond, T. J. Crystal structure of the nucleosome core particle at 2.8 Å resolution. *Nature* **1997**, *389* (6648), 251–260. DOI: 10.1038/38444.

(3) Bao, Y.; White, C. L.; Luger, K. Nucleosome Core Particles Containing a Poly(dA·dT) Sequence Element Exhibit a Locally Distorted DNA Structure. *Journal of Molecular Biology* **2006**, *361* (4), 617–624. DOI: https://doi.org/10.1016/j.jmb.2006.06.051.

(4) Ong, M. S.; Richmond, T. J.; Davey, C. A. DNA Stretching and Extreme Kinking in the Nucleosome Core. *Journal of Molecular Biology* **2007**, *368* (4), 1067–1074. DOI: https://doi.org/10.1016/j.jmb.2007.02.062.

(5) Wu, B.; Davey, C. A. Using Soft X-Rays for a Detailed Picture of Divalent Metal Binding in the Nucleosome. *Journal of Molecular Biology* **2010**, *398* (5), 633–640. DOI: https://doi.org/10.1016/j.jmb.2010.03.038.

(6) Vasudevan, D.; Chua, E. Y. D.; Davey, C. A. Crystal Structures of Nucleosome Core Particles Containing the '601' Strong Positioning Sequence. *Journal of Molecular Biology* **2010**, *403* (1), 1–10. DOI: https://doi.org/10.1016/j.jmb.2010.08.039.

(7) Mohideen, K.; Muhammad, R.; Davey, C. A. Perturbations in nucleosome structure from heavy metal association. *Nucleic Acids Research* **2010**, *38* (18), 6301–6311. DOI: 10.1093/nar/gkq420 (accessed 1/7/2026).

(8) Urahama, T.; Horikoshi, N.; Osakabe, A.; Tachiwana, H.; Kurumizaka, H. Structure of human nucleosome containing the testis-specific histone variant TSH2B. *Acta Crystallographica Section F* **2014**, *70* (4), 444–449. DOI: doi:10.1107/S2053230X14004695.

(9) Horikoshi, N.; Arimura, Y.; Taguchi, H.; Kurumizaka, H. Crystal structures of heterotypic nucleosomes containing histones H2A.Z and H2A. *Open Biology* **2016**, *6* (6), 160127. DOI: 10.1098/rsob.160127 (accessed 1/7/2026).

(10) Taguchi, H.; Xie, Y.; Horikoshi, N.; Maehara, K.; Harada, A.; Nogami, J.; Sato, K.; Arimura, Y.; Osakabe, A.; Kujirai, T.; et al. Crystal Structure and Characterization of Novel Human Histone H3 Variants, H3.6, H3.7, and H3.8. *Biochemistry* **2017**, *56* (16), 2184–2196. DOI: 10.1021/acs.biochem.6b01098.

(11) Arimura, Y.; Ikura, M.; Fujita, R.; Noda, M.; Kobayashi, W.; Horikoshi, N.; Sun, J.; Shi, L.; Kusakabe, M.; Harata, M.; et al. Cancer-associated mutations of histones H2B, H3.1 and H2A.Z.1 affect the structure and stability of the nucleosome. *Nucleic Acids Research* **2018**, *46* (19), 10007–10018. DOI: 10.1093/nar/gky661 (accessed 1/7/2026).

(12) Bilokapic, S.; Strauss, M.; Halic, M. Histone octamer rearranges to adapt to DNA unwrapping. *Nature Structural & Molecular Biology* **2018**, *25* (1), 101–108. DOI: 10.1038/s41594-017-0005-5.

(13) Bilokapic, S.; Strauss, M.; Halic, M. Structural rearrangements of the histone octamer translocate DNA. *Nature Communications* **2018**, *9* (1), 1330. DOI: 10.1038/s41467-018-03677-z.

(14) Hirai, S.; Tomimatsu, K.; Miyawaki-Kuwakado, A.; Takizawa, Y.; Komatsu, T.; Tachibana, T.; Fukushima, Y.; Takeda, Y.; Negishi, L.; Kujirai, T.; et al. Unusual nucleosome formation and transcriptome influence by the histone H3mm18 variant. *Nucleic Acids Research* **2022**, *50* (1), 72–91. DOI: 10.1093/nar/gkab1137 (accessed 1/7/2026).

(15) Arimura, Y.; Shih, R. M.; Froom, R.; Funabiki, H. Structural features of nucleosomes in interphase and metaphase chromosomes. *Molecular Cell* **2021**, *81* (21), 4377–4397.e4312. DOI: 10.1016/j.molcel.2021.08.010 (accessed 2026/01/06).
(16) Zheng, L.; Tsai, B.; Gao, N. Structural and mechanistic insights into the DNA glycosylase AAG-mediated base excision in nucleosome. *Cell Discovery* **2023**, *9* (1), 62. DOI: 10.1038/s41421-023-00560-0.
(17) Fukushima, Y.; Hatazawa, S.; Hirai, S.; Kujirai, T.; Ehara, H.; Sekine, S.-i.; Takizawa, Y.; Kurumizaka, H. Structural and biochemical analyses of the nucleosome containing Komagataella pastoris histones. *The Journal of Biochemistry* **2022**, *172* (2), 79–88. DOI: 10.1093/jb/mvac043 (accessed 1/7/2026).
(18) Wu, H.; Muñoz, E. N.; Hsieh, L. J.; Chio, U. S.; Gourdet, M. A.; Narlikar, G. J.; Cheng, Y. Reorientation of INO80 on hexasomes reveals basis for mechanistic versatility. *Science* **2023**, *381* (6655), 319–324. DOI: 10.1126/science.adf4197 (accessed 2026/01/06).
(19) Nozawa, K.; Takizawa, Y.; Pierrakeas, L.; Sogawa-Fujiwara, C.; Saikusa, K.; Akashi, S.; Luk, E.; Kurumizaka, H. Cryo–electron microscopy structure of the H3-H4 octasome: A nucleosome-like particle without histones H2A and H2B. *Proceedings of the National Academy of Sciences* **2022**, *119* (45), e2206542119. DOI: 10.1073/pnas.2206542119 (accessed 2026/01/06).
(20) White, C. L.; Suto, R. K.; Luger, K. Structure of the yeast nucleosome core particle reveals fundamental changes in internucleosome interactions. *The EMBO Journal* **2001**, *20* (18), 5207–5218. DOI: 10.1093/emboj/20.18.5207.
(21) Zhou, B.-R.; Jiang, J.; Feng, H.; Ghirlando, R.; Xiao, T. S.; Bai, Y. Structural Mechanisms of Nucleosome Recognition by Linker Histones. *Molecular Cell* **2015**, *59* (4), 628–638. DOI: 10.1016/j.molcel.2015.06.025 (accessed 2026/01/06).
(22) Wu, B.; Mohideen, K.; Vasudevan, D.; Davey, C. A. Structural Insight into the Sequence Dependence of Nucleosome Positioning. *Structure* **2010**, *18* (4), 528–536. DOI: 10.1016/j.str.2010.01.015 (accessed 2026/01/06).
